# Peripheral blood transcriptomic profiling of molecular mechanisms commonly regulated by binge drinking and placebo effects

**DOI:** 10.1038/s41598-024-56900-x

**Published:** 2024-05-10

**Authors:** Amol Carl Shetty, John Sivinski, Jessica Cornell, Carrie McCracken, Lisa Sadzewicz, Anup Mahurkar, Xing-Qun Wang, Luana Colloca, Weihong Lin, Nageswara Pilli, Maureen A. Kane, Chamindi Seneviratne

**Affiliations:** 1 Institute for Genome Sciences, University of Maryland School of Medicine, 670 W. Baltimore Street, Baltimore, MD 21201 USA; 2 Department of Pharmacology, University of Maryland School of Medicine, Baltimore, MD USA; 3 Department of Public Health Sciences, University of Virginia, Charlottesville, VA USA; 4 Department of Pain and Translational Symptom Science, Placebo Beyond Opinions (PBO) Center, University of Maryland School of Nursing, Baltimore, MD USA; 5 Department of Biological Sciences, University of Maryland, Baltimore County, Baltimore, MD USA; 6 Department of Pharmaceutical Sciences, University of Maryland School of Pharmacy, Baltimore, MD USA

**Keywords:** Biomarkers, Medical research, Molecular medicine, Psychiatric disorders

## Abstract

Molecular responses to alcohol consumption are dynamic, context-dependent, and arise from a complex interplay of biological and external factors. While many have studied genetic risk associated with drinking patterns, comprehensive studies identifying dynamic responses to pharmacologic and psychological/placebo effects underlying binge drinking are lacking. We investigated transcriptome-wide response to binge, medium, and placebo alcohol consumption by 17 healthy heavy social drinkers enrolled in a controlled, in-house, longitudinal study of up to 12 days. Using RNA-seq, we identified 251 and 13 differentially expressed genes (DEGs) in response to binge drinking and placebo, respectively. Eleven protein-coding DEGs had very large effect sizes in response to binge drinking (Cohen’s d > 1). Furthermore, binge dose significantly impacted the *Cytokine-cytokine receptor interaction pathway* (KEGG: hsa04060) across all experimental sequences. Placebo also impacted hsa04060, but only when administered following regular alcohol drinking sessions. Similarly, medium-dose and placebo commonly impacted KEGG pathways of *Systemic lupus erythematosus*, *Neutrophil extracellular trap formation*, and *Alcoholism* based on the sequence of drinking sessions. These findings together indicate the “dose-extending effects” of placebo at a molecular level. Furthermore, besides supporting alcohol dose-specific molecular changes, results suggest that the placebo effects may induce molecular responses within the same pathways regulated by alcohol.

## Introduction

Alcohol misuse is one of the leading risk factors for premature death and disability in the U.S. and worldwide^1^. Alcohol permeates virtually all tissues in the body, leading to multisystemic pathophysiological consequences linked to over 200 health conditions, creating a substantial global disease burden on society^1,2^. Pathophysiological consequences of alcohol use/misuse differ based on various characteristics of drinking, such as the amount, frequency, chronicity, and type of alcoholic beverage. Binge drinking is one of the most common patterns of alcohol misuse that increases the risk of developing alcohol use disorder (AUD) and other deleterious health consequences of alcohol misuse^2^. According to the 2019 National Survey on Drug Use and Health (NSDUH), approximately 24% of the U.S. population aged 12 years and older reported at least one binge drinking episode during the past month, surpassing the population diagnosed with AUD^3^.

Molecular responses to alcohol consumption patterns are governed by an individual’s genetic background, fine-tuned by numerous interacting environmental exposures (i.e., exposome) such as lifestyle or biological factors like epigenetic landscape, age, gender, and microbiome composition^4–10^. Thus, gene expression profiling, particularly at a transcriptome-wide level, may effectively capture a snapshot of dynamic and context-dependent molecular responses arising from simultaneous or concurrent interplay of biological and external factors. Identifying these dynamic mechanisms has the potential to further our understanding of pathological processes and biomarker development beyond genetic risk prediction. However, apart from the published animal or in vitro studies^11–13^, gene expression alterations by alcohol use/misuse are often conducted with pre-specified genes based on research hypotheses because of the complexity of specimen collection from living individuals and the prohibitive cost of transcriptome-wide analysis^14,15^. As an alternate strategy to mitigate these challenges, large population-based GWAS have been utilized recently to impute transcriptomic alterations resulting from varied alcohol consumption patterns^16^. While these large-scale datasets provide enhanced power to perform statistical associations, gene expression is a complex and non-linear product of many interacting factors as stated above. Hence, experimental studies that carefully control for the interacting exposome are essential to accurately model gene expression alterations in response to drinking behavior.

Furthermore, behavioral studies deciphering mechanisms underlying drinking behavior have demonstrated that at least some of the effects of alcohol are accounted for by the non-pharmacological component driven by psychological phenomena such as placebo effects. Placebo beverage administration studies conducted in laboratory settings have repeatedly demonstrated that the individuals presented with a placebo beverage often believed the drinks to contain alcohol^17,18^. Slower attentional processing^18^, subjective measures of intoxication^17^, and increased craving for alcohol^19^ seen in response to placebo alcohol administration further corroborate these observations. However, despite the long history of studies administering placebo alcohol beverages, it is still unclear whether the behavioral and subjective outcomes are induced by or stem from molecular-level changes similar to or distinct from the consumption of regular alcohol. Such knowledge may help develop novel tools to manipulate underlying molecular mechanisms to develop improved strategies for novel diagnostics and treatments for alcohol misuse.

Considering the above-detailed pharmacologic and non-pharmacologic mechanisms underlying drinking behavior, we hypothesized that the molecular responses to regular alcohol and placebo may overlap. In the present study, we performed a transcriptome-wide analysis to test our hypothesis, using peripheral blood samples collected from a cohort of binge drinkers enrolled in a cross-over human laboratory trial conducted in a controlled environment specifically designed to identify biomarkers of binge drinking while considering placebo effects underlying drinking behavior^20^. We investigated whether: (1) consumption of placebo and regular alcohol may regulate common molecular mechanisms, (2) binge-level alcohol consumption regulates specific pathways or molecular mechanisms not regulated by lower amounts of drinking, and (3) genes potentially regulated by pharmacological effects of alcohol.

## Results

**Figure 1:**
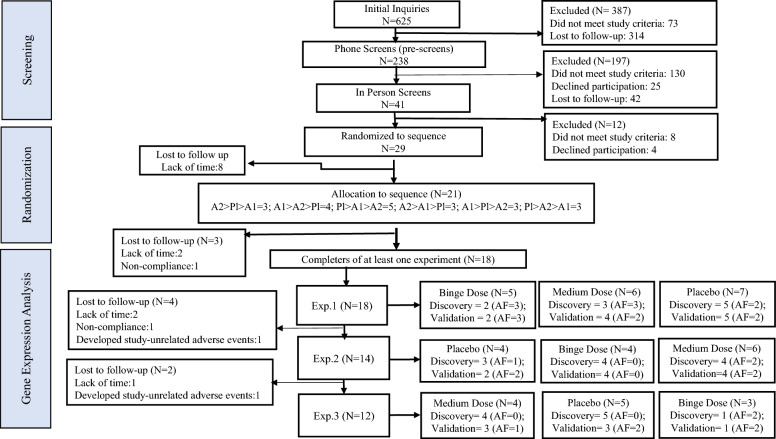
CONSORT Diagram of participants included in the transcriptomic analysis. Experiment.1–3 = three human laboratory experiments that were identical in every way except for the administered beverage dose; Discovery = discovery cohort; Validation = validation cohort; AF = RNA-seq and/or NanoString assay failures due to lack of availability of samples for the secondary analysis that passed quality control measures (see methods). Because of the small sample size of each cell, gene expression data collected from participants included in the six sequences shown in the “Allocation to Sequences” box were grouped further into three groups resulting in nine dose-by-experiment categories (the 9 boxes corresponding to Experiment.1–3). The groups were combined based on the allocation of beverage dose within each experiment regardless of the sequence of previous or subsequent doses. The flow of events across 3 × 3 Latin square study design are presented with the parent study.

We analyzed 62 total RNA samples using RNA-seq in the discovery cohort and validated a subset of 50 total RNA samples using NanoString nCounter assays based on the availability after RNA-seq analyses*.* All samples were derived from blood samples collected across 17 heavy social drinkers enrolled in an up to 12-day human laboratory trial (Fig. 1; Supplementary Table S1) who received three doses of alcoholic beverages within two-hour sessions, as described in the methods section. See Table 1 for participant demographic information by alcohol doses. The RNA-seq analysis yielded 28,294 *Ensembl* annotated genes (43.97% of all Ensembl annotated genes). All participants had zero breath alcohol concentration (BrAC) readings at admission to each 4-day in-house experiment, at the time of blood draws on D0 and D3, and immediately before the start of drinking sessions (See methods and Cornell et al^20^. for more details). As expected, the average BrAC readings taken directly following the 2-h drinking sessions differed significantly between dose groups both in the discovery (p = 6.86E-08) and the validation (p = 2.68E-06) cohorts (Table 1). The plasma ethyl glucuronide (EtG) levels were higher than the lower limit of quantification (LLOQ) in most participants tested 17–18 h after binge drinking (at the time of blood draw for transcriptomics) but undetectable (below LLOQ) after consuming the placebo dose.

**Table 1:** Demographics and study characteristics.

Characteristic	Total Population	Placebo	Medium	High	P_value
Discovery cohort					
Total No. of samples (Experiment-1; Experiment-2; Experiment-3)	62 (20;22;20)	26 (10;6;10)	22 (6;8;8)	14 (4;8;2)	ns
Age	27.50	27.38	29.09	30.70	ns
Gender (# of males; females (% male))	11; 5 (68.75%)	8; 5 (61.54%)	9; 2 (81.82%)	6; 1 (85.70%)	ns
Average BMI (SD)*	24.71 (3.69)	24.57 (3.62)	25.28 (3.56)	25.46 (3.58)	ns
Average AUDIT score (SD)	8.56 (2.48)	8.69 (2.66)	8.64 (2.16)	9.25 (2.12)	ns
Past 30 days drinking measures**					
Average drinks per drinking day (SD)	4.6 (1.9)	4.6 (2.0)	4.5 (1.2)	4.2 (1.5)	ns
Average number of binge episodes (SD)	7.3 (4.9)	7.7 (5.2)	7.9 (4.4)	8.6 (5.9)	ns
Past 90 days other drug use^#^					
Nicotine, Marijuana, Cocaine (No.)	2, 14, 0	2, 7, 0	0, 5, 0	1, 2, 0	
Average BrAC Levels^##^					
Pre-Dose, Day 3 (SD)	0.000 (0)	0.000 (0)	0.000 (0)	0.000 (0)	ns
Post-Dose, Day 3 (SD)	0.045 (0.051)	0.001 (0.002)	0.047 (0.021)	0.106 (0.051)	6.86E-08
EtG levels					
≥ LLOQ (%), Baseline	0 (0)	0 (0)	0 (0)	0 (0)	
0 > LLOQ (%), Baseline	2 (6.45)	1 (7.69)	0 (0)	1 (14.29)	
≥ LLOQ (%), Post-Dose	7 (22.58)	0 (0)	1 (9.09)	6 (85.71)	
0 > LLOQ (%), Post-Dose	3 (9.68)	0 (0)	2 (18.18)	1 (14.29)	
NanoString validation cohort					
Total No. of samples (Experiment.1; Experiment.2; Experiment.3)	50 (20;20;12)	18(8;4;6)	20 (8;8;4)	14 (4;8;2)	
Age	26.27	24.40	25.95	27.29	ns
Gender (# of males; females (% male))	73.33	7; 3 (67.00%)	9; 2 (80.00%)	5; 1 (85.71%)	
Average BMI (SD)*	25.56 (3.19)	25.66 (3.15)	25.95 (2.84)	24.79 (3.38)	ns
Average AUDIT score (SD)	8.71 (2.23)	8.55 (2.19)	8.11 (1.45)	8.57 (2.44)	ns
Past 30 days drinking measures**					
Average drinks per drinking day (SD)	4.9 (1.7)	4.8 (2.0)	5.00 (1.3)	4.6 (1.9)	ns
Average number of binge episodes (SD)	7.13 (4.3)	7.3 (5.2)	7.9 (4.5)	7.9 (6.3)	ns
Past 90 days other drug use^#^					
Nicotine, Marijuana, Cocaine (No.)	3, 14, 0	3, 9, 0	1, 1, 0	2, 4, 0	
Average BrAC Levels^##^					
Pre-Dose, Day 3 (SD)	0.000 (0)	0.000 (0)	0.000 (0)	0.000 (0)	ns
Post-Dose, Day 3 (SD)	0.046(0.047)	0.000(0)	0.059(0.034)	0.094(0.038)	7.43E-06
EtG levels					
≥ LLOQ (%), Baseline	1 (3.57)	0 (0)	1 (9.09)	0 (0)	
0 > LLOQ (%), Baseline	2 (7.14)	1 (10)	0 (0)	1 (14.29)	
≥ LLOQ (%), Post-Dose	8 (28.57)	0 (0)	3 (27.27)	5 (71.43)	
0 > LLOQ (%), Post-Dose	1 (3.57)	0 (0)	0 (0)	1 (14.29)	

ns = P > 0.05 for comparing the three groups and between placebo and medium or high dose alcohol groups. *P*-values were derived from the Fisher Exact test for categorical variables and the Kruskal Wallis test for continuous variables. *Calculated at the baseline of each experiment. **Calculated using standard drinks consumed 30 days before the initial in-person screen. ^#^Number of participants who used other drugs (excluding alcohol) 90 days before the initial in-person screen. ^##^Breath alcohol concentration (BrAC) reading units were g/210 L.

### Alcohol dose and experimental sequence had significant effects on WBC gene expression

**Figure 2:**
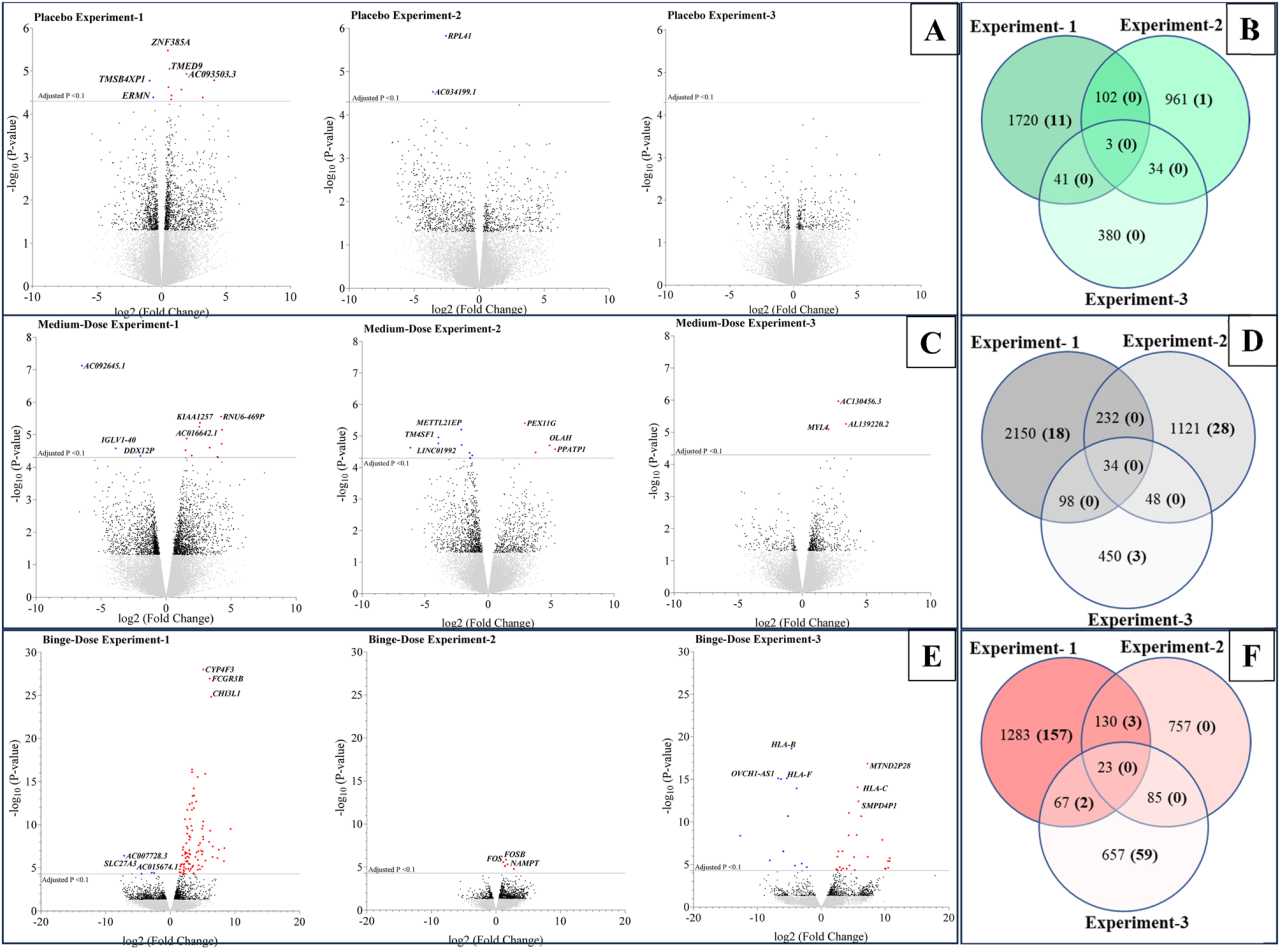
Distribution of detected DEGs within each experimental sequence stratified by the beverage doses assigned to an experiment across all participants. Volcano plots illustrate gene expression changes at D3 compared to D0 during the three experimental sequences in which the participants received a placebo (**A**), medium (**B**) and binge (**C**) alcohol doses. The y-axes represent the log transformed unadjusted *P*-values for the D3 vs D0 comparisons. The 10% thresholds for the FDR-adjusted *P*-values are indicated with horizontal dotted lines. Blue dots = downregulated DEGs; Red dots = upregulated DEGs; Black dots = nominally significant DEGs (i.e., *P*-value < 0.05 prior to adjustment for FDR). Gray dots = genes with statistically insignificant expression differences. The x- and y- axes are scaled differently between dose categories for legibility. The Venn diagrams (Fig. 2B,D,F) illustrate numerical representations of DEGs across experimental sequences within each dose category. The numbers within parentheses indicate DEGs that remained significant after adjustments for FDR.

One of the major findings of the parent study^20^ was that the expression levels of serotonin transporter (SERT) mRNA differed when the same dose was administered in experiments that differed in the sequence they occurred (i.e., when a given dose was assigned to experiment-1 vs. experiment-2 vs. experiment-3). Considering these significant sequence effects, here, we assessed the differential expression of genes (DEGs) between D0 and D3 within each dose-by-experiment group separately, rather than averaging expression levels across the three experiments for a given dose. Subsequently, we assessed the overall patterns of gene expression in response to the three tested doses by combining dose-by-experiment groups using a mixed-effects model. Supplementary Table S2 shows the differential expression of all genes (i.e., fold changes between D3 and D0) for the nine dose-by-experimental-sequence categories. DEGs varied across experiments and doses, with 12, 49, and 221 significant DEGs in at least one of the three placebo-, medium-, and binge-dose administered experiments, respectively, after adjustment for multiple comparisons (Fig. 2; Supplementary Table S2). No common DEGs were found in all three experiments where a specific dose was administered, indicating sequence effects of dose administration. However, four DEGs were found to be significantly upregulated in at least two of the three experiments in response to binge dose: *RNF182* (Ring Finger Protein 182) in experiment-1 and 3; *FOS* (Fos Proto-Oncogene, AP-1 Transcription Factor Subunit), *NAMPT* (Nicotinamide Phosphoribosyltransferase), and *DUSP1* (Dual Specificity Phosphatase 1) in experiment-1 and 2. The *CLC* (Charcot-Leyden Crystal Galectin) gene was commonly upregulated in binge dose and placebo-administered experiment-1 (Supplementary Fig. S1A). In contrast, *TMSB4XP1* (*TMSB4X* Pseudogene 1) was upregulated in binge dose-administered experiment-1 but marginally downregulated (i.e., fold-change < 1.5) in placebo- administered experiment-1.

When the data across experimental sequences for each dose were combined using a mixed-effects model, it was found that binge dose led to 54 significant DEGs, including the abovementioned genes *RNF182, FOS, NAMPT*, and *DUSP1*. On the other hand, there were only 17 significant DEGs for the medium dose and just one significant DEG for the placebo, which was *GMPR2* (Guanosine Monophosphate Reductase 2), in the combined analysis (refer to Supplementary Table S2).

### Alcohol dose-associated responses in gene expression, excluding potential placebo responses

The mixed-effects model analyses of RNA-seq data for binge-vs-placebo and medium-vs-placebo comparisons across all three experiments revealed 36 and 64 significant DEGs (FDR adjusted). The distribution of DEGs in the binge-vs-placebo comparison was 72.73% protein-coding, 18.18% processed pseudogenes, 6.06% antisense RNA genes, and 3.03% sense-intronic genes. The distribution of DEGs in the medium-vs-placebo comparison was 53.85% protein-coding, 11.54% pseudogenes, 19.23% anti-sense RNA genes, 5.77% sense-intronic, 3.85% lincRNA, 1.92% miRNA, and 3.85% other genes. *NanoString nCounter* assays were utilized to evaluate all significant protein-coding DEGs in both dose-vs-placebo comparisons (24 and 28, respectively, with no overlapping DEGs between comparisons). We considered a DEG as *validated* if results from both RNA-seq and NanoString were similar in statistical significance and direction of expression alterations for a given comparison—i.e., significantly upregulated, downregulated, or non-significant with both methods. Table 2 lists all protein-coding DEGs that passed validation with *NanoString* assays. These include, 16 of 52 tested DEGs in the binge-vs-placebo (30.77% validation rate) and 6 out of 52 tested DEGs in medium-vs-placebo comparisons (11.54% validation rate). The validation failures were due to (1) 23 DEGs that were selectively detected in the medium-vs-placebo comparison with RNA-seq were found to be significant in the binge-vs-placebo comparison when using NanoString assays, (2) 4 RNA-seq detected DEGs in binge-vs-placebo were not significant with *NanoString*, and (3) 22 RNA-seq-detected DEGs in the medium-vs-placebo were not significant with *NanoString*. It should also be noted that, compared to RNA-seq, *NanoString* assays detected more significant DEGs in the binge-vs-placebo (24 vs. 43 out of 52 tested genes) than in the medium-vs-placebo (28 vs. 17 out of 52 tested genes) and 15 DEGs were common to the two comparisons (only with *NanoString)*. The estimated Cohen’s d effect sizes ranged from 1.05 to 3.31 and 1.00–2.50 for the validated genes in the binge-vs-placebo and medium-vs-placebo comparisons, respectively, were very large (> 0.8) Therefore, these validated findings have not only statistical significance but also practical significance as well.

**Table 2:** Significantly differentially expressed protein-coding genes identified and validated in dose-vs-placebo comparisons across experiments.

Ensembl gene ID	Gene symbol	Binge vs. placebo (Discovery; N = 20)			Binge vs. placebo (Validation; N = 17)		Medium vs. placebo (Discovery; N = 24)			Medium vs. placebo (Validation; N = 21)	
		Beta value	P (FDR)	Cohen's d	Beta value	P (FDR)	Beta value	P (FDR)	Cohen's d	Beta value	P (FDR)
ENSG00000187627	*RGPD1*	**1.0106**	**7.03E-02**	**1.1732**	**0.0866**	**4.08E-14**	0.3811	3.60E-01	0.4844	-0.1572	3.95E-01
ENSG00000137571	*SLCO5A1*	**0.9290**	**8.01E-02**	**1.1347**	**0.8548**	**1.44E-21**	0.4059	3.70E-01	0.4764	0.3420	1.51E-01
ENSG00000171848	*RRM2*^21^	**1.1893**	**2.59E-02**	**3.0214**	**0.9023**	**3.59E-14**	0.5038	3.75E-01	0.5649	0.8073	9.76E-02
ENSG00000163378	*EOGT*	**0.4797**	**4.48E-02**	**2.4081**	**0.9424**	**1.42E-21**	-0.1993	4.24E-01	0.4116	0.1634	3.41E-01
ENSG00000145708	*CRHBP*^22–27^	**0.5990**	**3.26E-02**	**1.8810**	**0.8548**	**1.95E-15**	-0.2459	5.75E-01	0.3017	0.4279	9.76E-02
ENSG00000151136	*ABTB3*	**0.4897**	**6.34E-02**	**1.3813**	**0.5550**	**1.42E-21**	-0.1118	7.78E-01	0.1726	-0.2670	2.54E-01
ENSG00000204179	*PTPN20*	**1.3357**	**2.92E-02**	**3.3058**	**0.8548**	**1.42E-21**	0.2375	8.01E-01	0.1553	0.4342	9.76E-02
ENSG00000171992	*SYNPO*^28,29^	**-0.9983**	**8.38E-02**	**1.6797**	**-1.2110**	**1.42E-21**	-0.2211	8.09E-01	0.1515	0.3270	3.95E-01
ENSG00000139973	*SYT16*	**0.7339**	**6.34E-02**	**1.2980**	**0.8548**	**2.79E-15**	0.1070	8.29E-01	0.1453	0.4330	9.76E-02
ENSG00000189114	*BLOC1S3*	**-0.5092**	**8.38E-02**	**1.1328**	**-1.1900**	**8.36E-15**	-0.0835	8.39E-01	0.1517	0.2699	6.32E-01
ENSG00000188687	*SLC4A5*	**0.8192**	**8.67E-02**	**1.0464**	**0.2177**	**5.59E-21**	0.0535	9.08E-01	0.0809	0.4343	9.76E-02
ENSG00000130433	*CACNG6*	**0.6371**	**2.84E-02**	**2.1196**	**0.8548**	**1.21E-16**	0.0701	9.30E-01	0.0791	0.1966	7.18E-01
ENSG00000176014	*TUBB6*^30^	**-0.7074**	**7.03E-02**	**1.3060**	**-2.7153**	**3.13E-14**	0.0100	9.86E-01	0.0163	-0.0874	6.95E-01
ENSG00000078596	*ITM2A*	**0.5398**	**7.31E-02**	**1.8088**	**0.4149**	**7.13E-02**	0.1028	8.76E-01	0.1077	0.1003	8.07E-01
ENSG00000180537	*RNF182*	**1.8106**	**8.17E-04**	**2.2785**	**0.8078**	**7.32E-02**	-0.2726	5.61E-01	0.3095	0.2533	5.70E-01
ENSG00000080822	*CLDND1*	**0.4541**	**6.34E-02**	**1.3480**	**0.4383**	**8.71E-02**	0.0177	9.55E-01	0.0434	0.0910	7.18E-01
ENSG00000144596	*GRIP2*	0.4477	3.96E-01	0.6051	0.8548	9.47E-15	**0.8989**	**3.77E-02**	**1.5989**	**0.3808**	**9.76E-02**
ENSG00000151276	*MAGI1*	0.5759	4.74E-01	0.5176	0.8548	1.42E-21	**0.5131**	**9.20E-02**	**1.4001**	**0.3782**	**9.76E-02**
ENSG00000118298	*CA14*	0.4116	6.78E-01	0.3504	0.8548	1.42E-21	**0.7820**	**2.03E-03**	**2.5037**	**0.3724**	**9.78E-02**
ENSG00000188290	*HES4*	0.3331	7.73E-01	0.2653	0.4600	1.42E-21	**1.1497**	**2.98E-02**	**1.1036**	**0.4349**	**9.76E-02**
ENSG00000125966	*MMP24*^31^	0.0794	9.36E-01	0.0979	0.8548	1.31E-15	**0.3128**	**9.31E-02**	**1.3453**	**0.3869**	**9.76E-02**
ENSG00000170075	*GPR37L1*	0.0526	9.62E-01	0.0601	0.8548	1.44E-21	**0.8176**	**5.88E-02**	**1.0043**	**0.3666**	**9.91E-02**

N = number of sample pairs (i.e., pairs of baseline and post-treatment samples); ND = not detected. Bold values represent statistics for significant DEGs with similar directions within the discovery and validation cohorts. Citations are provided for the DEGs previously reported in human and/or animal studies to be associated with alcohol-related phenotypes.

### The *impacted* pathways overlapped across beverage doses

iPathwayGuide identified 341 pathways in the nine dose-by-sequence categories (Supplementary Table S3). Of the 384, ten pathways were statistically significantly (FDR adjusted) impacted by at least one of the three beverage doses, based on the enriched genes and their perturbations (Supplementary Table S4). Following four pathways were detected in multiple experiments for any beverage dose: The KEGG pathways *Systemic lupus erythematosus* (hsa05322), *Neutrophil extracellular trap formation* (hsa04613), *Alcoholism* (hsa05034), and *Cytokine-cytokine receptor interaction* pathway (hsa04060). As presented in Table 3, in response to the placebo, all four pathways had significant FDR-adjusted *P*-values when administered in the last double-blind experiment (i.e., Experiment-3; when the placebo was administered after completing two experiments where participants received regular alcohol at the binge and medium doses). The placebo impacted pathways of *Systemic lupus erythematosus*, *Neutrophil extracellular trap formation*, and *Alcoholism* via upregulation of seven out of the 80 genes in *H2A, H2B, H3*, and *H4*, gene classes (*H2BC21, H2BC5, H2AC16, H2BC4, H2AC8, H4C15, and H3C13*) expressed in the nucleosome (GO:0,000,786; *P*-value (FDR) = 0.002). Upregulated genes *H2BC21, H2BC5*, and *H2AC16* interacted to form a shared network through which the placebo putatively impacted all three pathways. Within the putative network, *H2BC21* and *H2BC5* were predicted to interact via activation/catalyzation, while upregulated genes *H2BC5* and *H2AC16* were predicted to interact with each other by binding their protein products. Ten DEGs, including the seven mentioned above, were found to have protein heterodimerization activity (GO:0,046,982; *P*-value (FDR) = 0.023) out of 259 assessed genes. The *H2A, H2B, H3*, and *H4* gene classes were upregulated in response to the binge dose in experiment-3 (21 genes) and medium dose in experiment-1 (20 genes). Notably, unlike the placebo that altered pathways hsa05322, hsa04613, and hsa05034 exclusively via the upregulation of *H2A, H2B, H3*, and *H4* gene classes, binge and medium doses impacted additional gene classes contributing to the upregulation of hsa05322, hsa04613, and hsa05034 KEGG pathways.

**Table 3:** Significantly altered pathways detected within each 4-day experiment in response to beverage doses.

Pathway Name (KEGG ID)	Dose	Experiment-1				Experiment-2				Experiment-3			
		Count (DE/All)	Rank	Unadjusted	FDR Adjusted	Count (DE/All)	Rank	Unadjusted	FDR Adjusted	Count (DE/All)	**Rank**	**Unadjusted**	**FDR Adjusted**
				*P*-value	*P*-value			*P*-value	*P*-value			*P*-value	*P*-value
Systemic lupus erythematosus (hsa05322)	Binge	9/94	37	0.3171	0.8260	4/94	34	0.1914	0.8042	23/94	3	*4.60E-06*	***0.0004***
	Medium	23/97	3	*0.0003*	***0.0372***	17/98	1	*2.7646E-05*	***0.0073***	8/98	1	*0.0038*	0.6920
	Placebo	10/100	5	*0.0152*	0.8386	5/101	13	0.0524	0.8718	7/101	1	*0.0002*	***0.0251***
Neutrophil extracellular trap formation (hsa04613)	Binge	17/157	11	*0.0200*	0.5607	5/157	49	0.7031	0.8980	24/157	1	*8.56E-07*	***0.0001***
	Medium	35/160	1	*8.39E-06*	***0.0018***	19/161	2	*0.0003*	***0.0456***	9/161	2	*0.0353*	0.6920
	Placebo	11/164	11	0.0688	0.9808	3/165	19	0.4896	0.8718	8/165	2	*0.0004*	***0.0318***
Cytokine-cytokine receptor interaction (hsa04060)	Binge	27/199	1	0.0004	***0.0987***	18/199	2	*0.0008*	***0.0953***	7/199	38	0.3566	0.8035
	Medium	26/207	29	0.2997	0.8852	19/207	7	*0.0299*	0.7199	7/207	7	0.3464	0.7626
	Placebo	16/203	7	0.0250	0.9808	11/203	14	0.1171	0.8718	4/203	4	*0.0014*	***0.0538***
Alcoholism (hsa05034)	Binge	15/141	23	0.0990	0.8133	3/140	41	0.3866	0.8228	22/140	2	*9.40E-07*	***0.0001***
	Medium	27/143	4	*0.0006*	***0.0494***	18/143	3	*0.0027*	0.2340	8/143	3	*0.0211*	0.6920
	Placebo	12/148	9	*0.0342*	0.9808	3/148	23	0.9537	0.9826	7/148	3	*0.0009*	***0.0448***

Italicized values represent significant *P*-values before FDR adjustments, and bold italicized values represent *P*-values that remained significant after adjustments for FDR.

**Figure 3:**
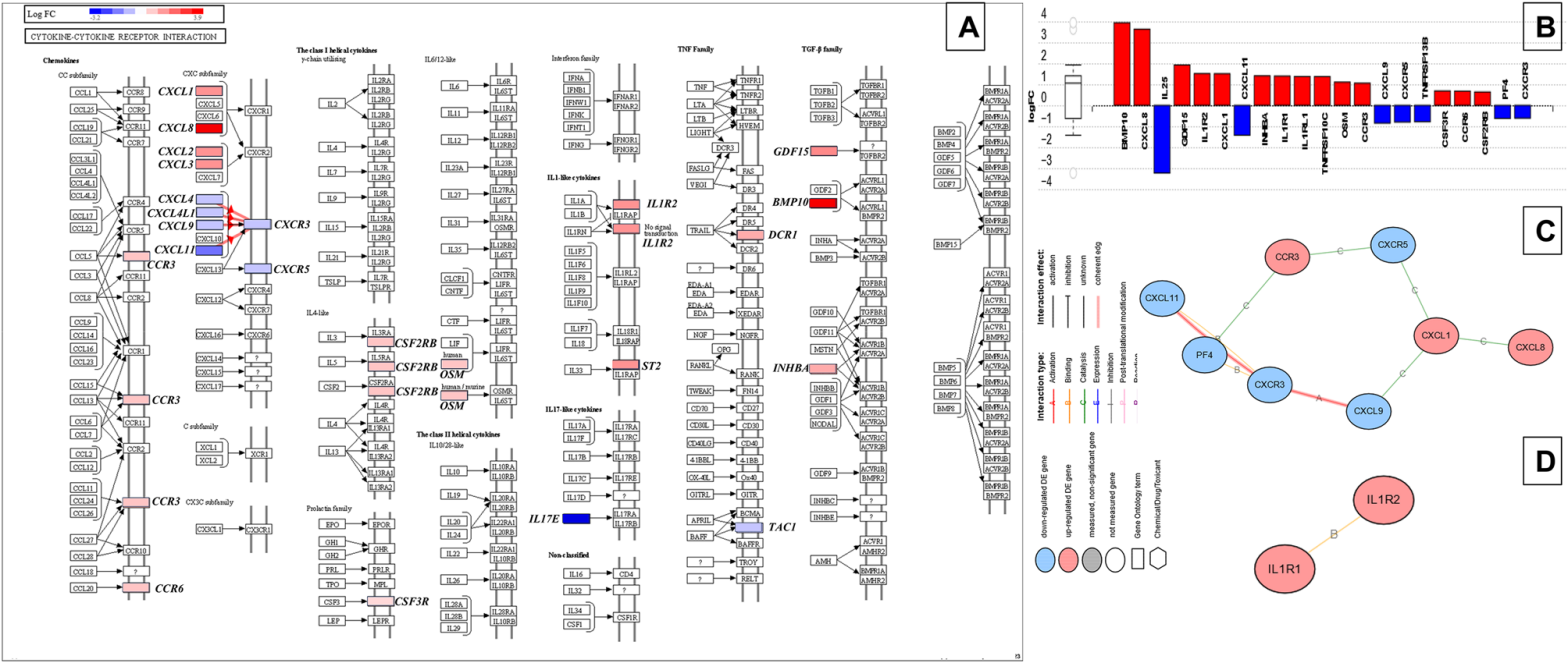
Binge drinking and placebo alcohol were associated with alterations in the Cytokine-cytokine receptor interaction pathway (hsa04060). Upregulated genes are shown in red, and downregulated genes are shown in blue in all Figures. Gene symbols for DEGs within the pathway are labeled outside corresponding boxes for legibility. **(A)** The pathway diagram overlayed with the computed perturbation of each gene within pathway hsa04060 in the mixed model analysis of all binge-dose administered visits. The perturbations account for the genes' measured fold changes and the accumulated perturbations propagated from upstream genes (accumulation). The highest negative perturbation is in dark blue, while the highest positive perturbation is in dark red. The legend describes the values of the gradient. For legibility, one gene may be represented in multiple locations in the diagram, and one box may represent multiple genes in the same gene family. A gene is highlighted in all locations it occurs in the diagram. The color corresponding to the gene with the highest absolute perturbation is displayed for each gene family. Red lines with arrows indicate the sequence of steps and the direction of the signal propagation (i.e., coherent cascades) for which the observed expression changes agree with the expected changes. **(B)** Measured expression levels of genes within pathway hsa04060 that correspond to Fig. 3A ranked based on their absolute log fold change values. The box and whisker plot on the left summarizes the distribution of all the differentially expressed genes in pathway hsa04060. The box represents the 1st quartile, the median, and the 3rd quartile, while circles represent the outliers. Putative mechanisms (Fig. 3C and 3D) through which binge dose may act on the genes measured to be differentially expressed in pathway hsa04060. Figure 3C represents gene-by-gene interactions within the cascade illustrated with red arrows in Fig. 3A. Figure 3D represents upregulated IL-1 and IL-2 interactions within IL-1–type cytokine sub-pathway, suggesting a proinflammatory response.

The Cytokine-cytokine receptor interaction pathway was the most significantly impacted in response to the binge dose (in experiment-1, experiment-2, and combined analysis across all binge-dose administered experiments; *P*-value (FDR) = 0.004; Fig. 2). Figure 2A,B illustrate the 21 out of 201 DEGs enriched in the pathway and the propagation of signals (red lines) from extracellular chemokines *CXC* subfamily that contributed to the significant pathway impact. The sequences of pathway signals for measured expression levels in response to binge dose were consistent with the computed series of events for genes shown in Fig. 2C,D, inferring two putative networks within the Cytokine-cytokine receptor interaction pathway. Conversely, in response to the placebo, this pathway was impacted only during experiment-3 via four DEGs (*IL2, IL11, MSTN, and CXCR6*; Supplementary Table S3), and the measured expression levels were not consistent with the computed sequence of events (i.e., gene-by-gene interactions resulting in putative mechanisms were not identified within the pathway), implying a relatively weaker impact by placebo alone. The *iPathwayguide* analyses did not identify any significantly impacted pathways when placebo responses were subtracted from binge- and medium-dose responses (i.e., in binge-vs-placebo and medium-vs-placebo comparisons) (Fig. 3).

## Discussion

The patterns of alcohol consumption are a significant determinant of alcohol-related mortality risk and progression from light drinking to AUD^32^. Identifying the altered molecular mechanisms can help uncover novel treatment targets or develop prognostic biomarkers of alcohol-related pathology across the spectrum of drinking behaviors. Here, we conducted a transcriptome-wide analysis of gene expression alterations in response to binge drinking and underlying placebo effects in heavy drinkers who did not meet the criteria for AUD. Our results provide initial evidence of changes in peripheral blood WBC RNA and molecular pathways, that could potentially develop as biomarkers of responses to binge drinking, as well as placebo responses underlying drinking behaviors.

We employed two statistical models that assessed dose-specific changes within- and across experimental sequences, accounting for underlying placebo responses. These analyses lead to three main observations: First, our findings suggest that the order in which the beverages were administered significantly influenced the number of DEGs, with the first sequential experiment having the most DEGs compared to the subsequent experiments irrespective of the strength of alcohol. While these expression patterns of individual genes align well with our previous report that focused on a single gene—i.e., SERT mRNA expression levels^20^, they appear to contradict findings at the level of molecular pathways, specifically in response to placebo that showed significant alterations in the last, as opposed to the first sequential experiment. These differences likely occurred as the magnitude and direction of expression levels for individual DEGs varied across doses and experimental sequences, which may have resulted in differing cumulative effects that contributed to pathway level expression changes that are detailed below. Our second observation conforms with many *in invitro* and animal studies that demonstrated a positive correlation between alcohol dose and molecular responses. We detected 251 and 66 unique DEGs significantly (i.e., after FDR adjustments) altered by binge and medium doses, respectively (Supplementary Table S2). Third observation was that, when the placebo responses were subtracted from binge and medium doses to gain a preliminary understanding of the embedded placebo effects, the number of binge-dose-responsive DEGs reduced drastically (from 251 to 86 DEGs) while there was a slight increase in the medium-dose-responsive DEGs (From 66 to 86 DEGs). These findings raise the question whether the placebo responses are more robust with more severe drinking. Earlier studies have shown that expectancies of positive effects of alcohol predicted greater frequency and quantity of alcohol intake^33,34^. As we haven’t systematically assessed expectancies in this study, it is difficult to ascertain whether the converse—i.e., exposure to a higher dose of alcohol leads to greater expectancies or whether expectancies serve as an intermediate phenotype that is directly associated with dose-specific gene expression changes. On the other hand, the double-blind study design and the anecdotal feedback given by the participants during experimental sessions about the type of beverage they received argue for these possibilities. In fact, published behavioral studies have indeed demonstrated a substantial placebo component underlying drinking. Nonetheless, results from dose-vs-placebo comparisons and the 66 DEGs and pathway changes detected in the placebo arm suggest a significant contribution by placebo effects to the expression of these genes, and add to the limited data supporting a biological basis of placebo responses in drinking. Further testing is needed to harness the precise mechanisms.

Another strength of our study is that we assessed the validity of our gene-level findings using targeted NanoString *nCounter* assays that utilize an absolute quantification method. We tested a subset of 52 protein-coding genes of the 172 medium- and binge-dose responsive DEGs after subtracting the embedded placebo responses (Table 2). The concordance of results between RNA-seq and NanoString assays was relatively low (i.e., < 30%) compared to the published methodological validation studies comparing RNAseq with NanoString technologies. The results section above has detailed several potential reasons for these discrepancies. Nonetheless, the validated DEGs had very large effect sizes (Cohen’s d > 0.8) for the associations with alcohol’s pharmacologic effects as assessed by comparing medium (6 DEGs) and binge (15 DEGs) doses with placebo (Table 2). As a measure of practical significance, these very large effect sizes indicated how promising these validated findings were. Eleven of the 15 binge-dose-selective and five of the six medium-dose-selective validated DEGs are novel additions to the genes associated with the dose-specific effects of alcohol.

There are three key findings from our pathway-level analysis testing the *impact* of dose-specific alterations of genes on molecular mechanisms: (1) placebo responses significantly impacted four pathways that were also impacted by medium and binge doses, albeit via fewer genes than that were altered by regular alcohol consumption; (2) the impact of the placebo responses on the four pathways was significant only when it was administered in the last sequentially scheduled experiments following exposure to medium and binge doses, implying a molecular-level mechanism similar to the *dose-extending effects of placebos* described in behavioral and pharmacologic studies; and (3) none of the identified pathways remained significant when placebo responses were subtracted from pathway analyses within medium and binge dose groups. These findings together suggest a potentially significant placebo component (representing the context of beliefs) altering molecular mechanisms underlying drinking behavior. It is possible that the placebo's ability to extend the dose effects of alcohol may be due in part to participants being conditioned by prior experiments involving regular alcohol intake, even though they were accustomed to heavy drinking. We investigated the alternate possibility of carryover effects of alcohol on gene expression in the preceding experiment, but we ruled it out for several reasons: (1) cross-over study design that included long enough washout periods; (2) we normalized all post-drinking expression data within each experiment against its own baseline; and, (3) objective measurements of plasma EtG levels and BrAC readings at each experiment's baseline confirmed alcohol abstinence. In fact, placebos have been shown to successfully extend the analgesic effects of opioids in pain management^35,36^. Studies exploring the role of placebo conditioning on molecular pathways underlying pain disorders have indeed demonstrated that plasma interleukin (IL)-2 was reduced in placebo-conditioned immunosuppression (60, 61). Whether the downregulation of IL-2 detected in our study leads to clinically appreciable immune suppression remains to be explored using a more comprehensive approach. Overall, our findings suggest similar molecular mechanisms underlying drinking behavior.

There are a few caveats to be considered when interpreting our findings. First, we had a modest sample size vulnerable to imbalances in genetic variation confounding gene expression (not assessed in the present analysis) between dose-by-experiment categories. However, unlike in a parallel group design, the cross-over design randomly assigned participants to three sequences, each of which was a 4-day inhouse experiment that allowed each participant to have their own baseline gene expression measurements before a beverage dose. Consequently, we could use baseline-adjusted gene expression counts in between-group analyses, improving confidence in our results. Furthermore, as previously reported^20^, the study was conducted in a highly controlled setting maintaining environmental factors constant across participants and experiments as much as possible. The second caveat was that, because of the prohibitively expensive cost, we limited the validation step to assessing transcriptome wide-significant protein-coding DEGs detected in the comparisons between regular alcohol doses with placebo. Therefore, the non-coding DEGs shown in dose-vs-placebo comparisons and the pathway associations in response to dose-by-experiment categories should be interpreted cautiously. Third, the subjective effects of placebo alcohol administration were not assessed systematically, limiting our ability to directly correlate behavioral constructs with molecular alterations. Further, we used the commercially available non-alcoholic beer (O’Douls) as the blinded placebo, which had a similar consistency and aroma to the regular alcoholic beverage administered in the study. While this approach aligns with published behavioral studies that explored placebo effects underlying drinking^17^, it is still possible that the non-alcoholic contents may have contributed to the detected gene expression alterations directly or indirectly acting upon other physiological systems. Even if this was the case, the DEGs that we saw to be associated with the pharmacological effects of regular alcohol would likely have survived, as the main difference between the two beverage types was the content of alcohol which was negligible in the non-alcoholic beer. Whether the DEGs associated with the non-alcoholic beverage were, in fact, due to actual placebo effects needs further exploration using a beverage-free arm akin to no-treatment arms in placebo studies^35^ and by incorporating newer technologies such as the virtual reality that could simulate drinking environments. Despite these shortcomings, our study presents the first transcriptome-wide assessment of placebo alcohol administration, providing a framework for more structured studies in the future.

In conclusion, we present initial clues of molecular mechanisms commonly regulated by the pharmacological effects of alcohol and placebo effects underlying drinking. Critical next steps would be exploring whether the identified molecular mechanisms can be optimized with improved study paradigms and applying more sensitive molecular techniques such as single-cell transcriptomics or profiling plasma cell-free transcriptome to uncover non-invasive (i.e., peripheral) biomarkers for identifying novel treatment targets and diagnostics.

## Methods

### Participants

This study analyzed a subset of a more extensive parent study that sought to validate SERT mRNA as a quantitative biomarker of binge alcohol consumption in the absence of AUD and pharmacological or behavioral treatments^20^ (ClinicalTrials.gov Identifier: NCT02315885). Healthy adult volunteers of Hispanic or non-Hispanic European ancestry were included if they had a binge drinking episode of five or more (men) or four or more (women) standard drinks in one sitting in the past 30 days (one standard drink = 14 g of pure alcohol) (Robbins et al., 2020)). Participants were enrolled at the University of Virginia and the University of Maryland School of Medicine from 2013 to 2019. Written informed consent was obtained from all participants prior to starting study procedures and all methods were carried out in accordance with relevant guidelines and regulations. All experimental procedures were conducted in accordance with the protocols approved by the institutional review boards at each institution (Institutional Review Board for Health Sciences Research (IRB-HSR) of University of Virginia, and the IRB of University of Maryland, Baltimore) and monitored by a three-member data and safety monitoring board (DSMB). A detailed list of inclusion and exclusion criteria and the consenting process were reported previously^20^.

### Study design

**Figure 4:**
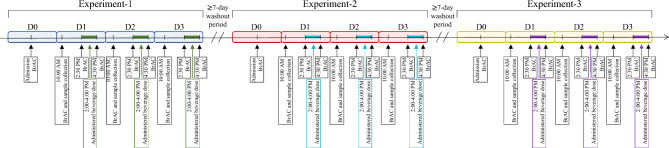
Scheduled dose administration, BrAC readings, and sample collection for all experiments. As detailed previously, the three doses were randomly assigned to experiment.1–3^20^. Experiment.1–3 = sequentially conducted experiments 1 through 3. D0-3 = session days within each experiment. BrAC = breath alcohol concentration. Colored horizontal bars within D1-3 represent 2 h-drinking sessions. The arrow across experiments 1–3 indicates the direction of the experimental sequence.

Enrolled participants were randomized to receive three alcoholic beverage doses in a double-blind human laboratory study. The three doses were: (1) placebo, (2) 0.5 g/kg (men) or 0.4 g/kg (women) alcohol (medium-dose), and (3) 1 g/kg (men) or 0.9 g/kg (women) alcohol that corresponds to binge drinking conditions (binge-dose). Each beverage dose was given in separate but otherwise identical four-day-long experiments. The beverage-free starting day of each experiment was used as the baseline (D0), and the remaining three days within an experiment consisted of 2-h sessions once daily where participants received an identical dose. Beverage dose differed between the three experiments. Therefore, each participant was scheduled to receive three beverage doses in the three separate experiments, each consisting of three once-daily sessions of an identical dose, randomly assigned for that specific experiment. A minimum of seven days living in the community separated experiments allowing for washout periods of more than five half-lives (t_1/2_) of median human cell mRNA (t1/2 = 10 h)^37^ and alcohol (t1/2 = 4-5 h)^38^. Participants were closely monitored and prohibited from eating or drinking anything not part of the standardized protocol. A total of 24 mL of whole blood was collected daily from each participant using collection tubes containing acid citrate dextrose (ACD) buffer (Vacutainer®, Becton–Dickinson, Franklin Lakes, NJ) at D0 and 17.5 to 18 h after the end of each drinking session to allow for late-onset gene expression alterations^39–41^. See Fig. 4 for an overview of the procedures.

### Measures of known direct biomarkers of alcohol consumption

We measured two known biomarkers, breath alcohol concentration (BrAC) and plasma ethyl glucuronide (EtG), at each session day to objectively assess the presence of alcohol and its metabolite EtG at D0, pre-, and post-drinking. The BrAC measurements were collected at admission, before and after dosing sessions, and before blood sample collection for RNA and EtG analyses. The WBC and plasma derived from the same whole blood samples were used for total RNA extractions and the detection of EtG, respectively. The EtG levels were determined using the liquid chromatography-tandem mass spectrometric method^20,42,43^.

### Total RNA isolation and sequencing

We analyzed white blood cell (WBC) samples isolated from whole blood collected at D0 and on the final day of each experiment (i.e., after three daily drinking sessions (D3)). According to the manufacturer's guidelines, total RNA was extracted using Macherey–Nagel’s NucleoSpin® miRNA kit and RNA/DNA Buffer kit (Takara Bio USA, Inc. Doral, Fl, USA). The quality was tested using an Agilent bioanalyzer system, and all samples with an RNA integrity number (RIN) greater than seven were selected for sequencing. Paired-end (PE) libraries were prepared and sequenced on the Illumina HiSeq4000 platform (Illumina, Inc., San Diego, CA) at a sequencing depth of 150 million reads at 100 bp PE length sequences.

### Sequencing data analyses

The raw sequence reads generated for each sample were analyzed using the CAVERN analysis pipeline^44^ and assessed quality with the FastQC toolkit for downstream analyses. The reads were aligned with the human reference genome GRCh38 (Ensembl repository) using fast splice-aware aligner HISAT2^45^ under default parameters to generate the alignment BAM files. The read alignments were assessed to compute gene expression counts with the HTSeq count tool^46^ and the human reference annotation (GRCh38). The raw read counts were normalized for library size and dispersion of gene expression and utilized in downstream analyses at the individual gene and pathway levels.

### Influence of beverage doses on individual genes

In the parent study, we detected significant sequence effects on SERT mRNA expression levels when the same beverage dose was administered in experiment-1, experiment-2, and experiment-3^20^. Hence, we separately assessed the differential expression of genes (DEGs) between D0 and D3 within each dose-by-experiment group using DESeq2 rather than averaging expression levels across the three experiments for a given dose. We analyzed DEGs for the following nine conditions comparing expression levels between D0 and D3 paired data from each individual: (1) placebo administered in experiment-1; (2) medium-dose in experiment-1; (3) binge-dose in experiment-1; (4) placebo in experiment-2; (5) medium-dose in experiment-2; (6) binge-dose in experiment-2; (7) placebo in experiment-3; (8) medium-dose in experiment-3; (9) binge-dose in experiment-3. The *P*-values were generated using the Wald test implemented in DESeq2 and then corrected for multiple hypothesis testing with the Benjamini–Hochberg correction method^47^. Next, we explored the effects of each dose on baseline-adjusted fold-changes across all three experiments where a specific dose was administered by combining experiment-specific data using a generalized linear mixed-effects models. We also performed a variance partition analysis that identified ‘variation across individuals’ as the major driver of variance in our dataset (Supplementary Fig. S2). A median of ~ 37% of the variation in expression was detected after correcting for gender, time, dose, and experiment. Hence, our linear mixed-effects model used Individual as a random effect and dose and experimental sequence as fixed effects. Additionally, genes on the sex chromosomes were filtered out from our results due to the imbalance in the gender of the participants in our cohort.

To distinguish between alcohol’s pharmacological and potential placebo effects, we separately compared binge and medium doses to placebo when administered in experiment-1, experiment-2, and experiment-3. Next, we utilized a generalized linear mixed-effects model to combine the effects of dose versus placebo on gene expression levels across the three experimental sequences. The model parameters were similar to the above-mentioned exploratory analysis conducted within each dose group. We used a 10% false discovery rate (FDR) and a minimum absolute log2 fold-change of 0.6 to determine the significant DEGs between conditions in all comparisons.

### Pathway analyses

We used iPathwayGuide (Advaita Bioinformatics, Plymouth Michigan, USA) to analyze the impact of three beverage doses on molecular pathways. This software identifies the “*impact*” of DEGs within pathways defined by the Kyoto Encyclopedia of Genes and Genomes (KEGG; Release 100.0 + /11–12, Nov 21)^48,49^ based on (1) over-representation of DEGs within a pathway and (2) perturbation propagating along the pathway topology. Perturbations were computed using gene ontologies obtained from the Gene Ontology Consortium database (2021-Nov4)^50^, a network of regulatory relations from BioGRID, and the Biological General Repository for Interaction Datasets v4.4.203. Oct. 25th, 2021^51^. To obtain distinct pathway-specific *P*-values for the overall “impact” of all DEGs on a specific pathway, we utilized Fisher’s method to merge two independent probability values, pORA (over-representation *P*-value) and pAcc (total accumulation *P*-value). Putative mechanisms were inferred employing *Advaita Knowledge Base* (AKB v1910, www.advaitabio.com) when measured gene expression changes were consistent with the computed sequence of events within a pathway. As listed above, we conducted pathway analyses on all the contrasts examined for individual gene-level effects. This amounted to 20 analyses (nine analyses for dose effects within each experiment, six analyses for dose-vs-placebo effects within each experiment, three analyses for each dose across its three experiments, and two analyses for each dose dose-vs-placebo effects across its three experiments). All genes with measured expression levels were included in the iPathwayGuide input files. Pathways were identified based on DEGs not adjusted for FDR at p < 0.05 statistical threshold. The pathway-specific *P*-values were subsequently adjusted for an FDR of 10% to determine if a pathway was statistically significant.

To control for type I error rate due to multiple testing, we adjusted the *P*-values across all analyses, using a false discovery rate (FDR) with a q-value threshold of 0.1, indicating significance, as suggested by Van den Oord and Sullivan^52^. To quantify the effect size of the difference between two groups, Cohen’s d effect size was calculated as a measure of practical significance.

### Gene Expression validation with NanoString assays

Protein-coding genes differentially expressed between regular alcohol (binge or medium doses) and placebo with RNA-seq were validated using customized *NanoString nCounter* assays (*NanoString* Technologies, Seattle, WA, USA). The validation sample included 50 pre- and post-treatment total RNA samples across 15 healthy heavy social drinkers with a 68% overlap with the discovery cohort. The NanoString code set additionally included two housekeeping genes (*GAPDH* and *MPP1*) for data normalization and spike-in positive (N = 6) and negative (N = 8) controls to set a minimum threshold count above background for data analyses. We used capture and reporter probe sets that were hybridized with at least 100 ng of total RNA for each sample. The hybridization reactions were processed on a nCounter Prep Station (Version 1, *NanoString* Technologies, Seattle, WA, USA). This step removed excess capture and reporter probes and immobilized and aligned hybridized complexes for binding to cartridges. Following the manufacturer’s guidelines, a single molecular counting image was created, and data was collected.

### NanoString data analysis

The mRNA raw data counts were analyzed using nSolver™ Analysis Software (Version 4.0; NanoString Technologies, Seattle, WA, USA). The six negative controls were employed to perform background thresholding. In contrast, positive controls were used to perform technical normalization to rectify any lane-by-lane variability caused by variation in hybridization or binding. Post technical normalization, the NanoString readings were analyzed using identical generalized linear mixed-effects model to assess expression levels between binge-dose vs. placebo and medium-dose vs. placebo beverage administrations.

### Supplementary Information

Supplementary Figure S1.

Supplementary Figure S2.

Supplementary Table S1.

Supplementary Table S2.

Supplementary Table S3.

Supplementary Table S4.

## Data Availability

Complete transcriptomic data used to support the findings of this study has been deposited in NIH Gene Expression Omnibus (GEO) repository (https://www.ncbi.nlm.nih.gov/geo/query/acc.cgi?acc=GSE232408) and embargoed until results from complete datasets are in press. GEO token: czopciuurxgbdgb.
