# Supplementary material for: Peripheral blood transcriptomic profiling of molecular mechanisms commonly regulated by binge drinking and placebo effects
Source: Sci Rep. 2024 May 10;14:10733. doi: 10.1038/s41598-024-56900-x (PMC11087488; doi:10.1038/s41598-024-56900-x)
Supplement: Supplementary file 1 — Supplementary Figure S1. [file 41598_2024_56900_MOESM1_ESM.pptx]

## Slide 1
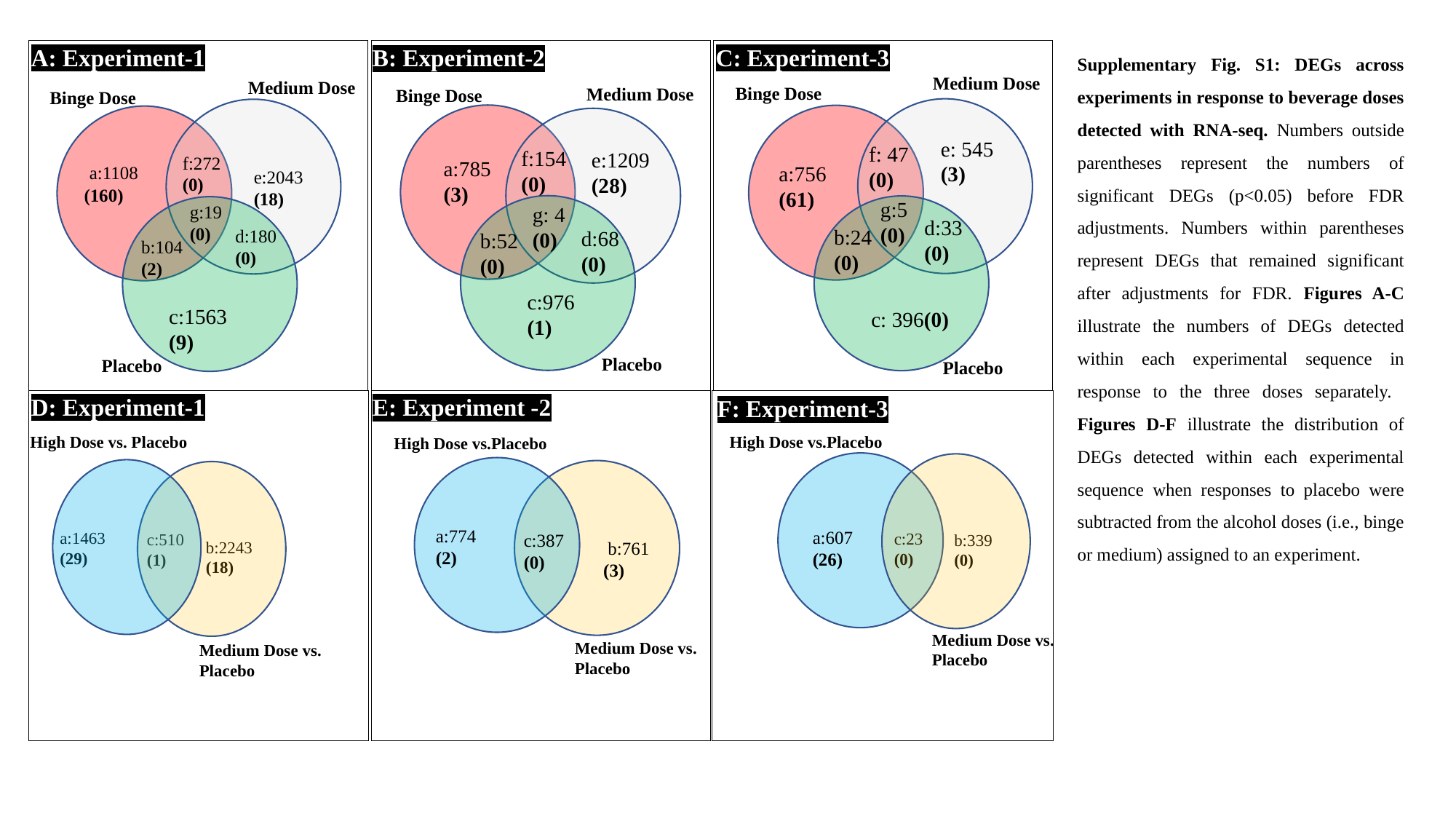

A: Experiment-1
C: Experiment-3
B: Experiment-2
D: Experiment-1
E: Experiment -2
F: Experiment-3
High Dose vs. Placebo
High Dose vs.Placebo
a:774 (2)
c:387 (0)
 b:761 (3)
Medium Dose vs. Placebo
a:607 (26)
a:1463 (29)
c:23 (0)
c:510 (1)
b:339 (0)
b:2243 (18)
Medium Dose vs. Placebo
High Dose vs.Placebo
Medium Dose vs. Placebo
Medium Dose
Medium Dose
Binge Dose
e: 545 (3)
f: 47 (0)
a:756 (61)
g:5 (0)
d:33 (0)
b:24 (0)
c: 396(0)
Placebo
Medium Dose
Binge Dose
f:154 (0)
e:1209 (28)
a:785 (3)
g: 4 (0)
d:68 (0)
b:52 (0)
c:976 (1)
Placebo
Binge Dose
f:272 (0)
 a:1108 (160)
e:2043 (18)
g:19 (0)
d:180 (0)
b:104 (2)
c:1563 (9)
Placebo
Supplementary Fig. S1: DEGs across experiments in response to beverage doses detected with RNA-seq. Numbers outside parentheses represent the numbers of significant DEGs (p<0.05) before FDR adjustments. Numbers within parentheses represent DEGs that remained significant after adjustments for FDR. Figures A-C illustrate the numbers of DEGs detected within each experimental sequence in response to the three doses separately. Figures D-F illustrate the distribution of DEGs detected within each experimental sequence when responses to placebo were subtracted from the alcohol doses (i.e., binge or medium) assigned to an experiment.
