# Supplementary material for: Peripheral blood transcriptomic profiling of molecular mechanisms commonly regulated by binge drinking and placebo effects
Source: Sci Rep. 2024 May 10;14:10733. doi: 10.1038/s41598-024-56900-x (PMC11087488; doi:10.1038/s41598-024-56900-x)
Supplement: Supplementary file 2 — Supplementary Figure S2. [file 41598_2024_56900_MOESM2_ESM.pptx]

## Slide 1
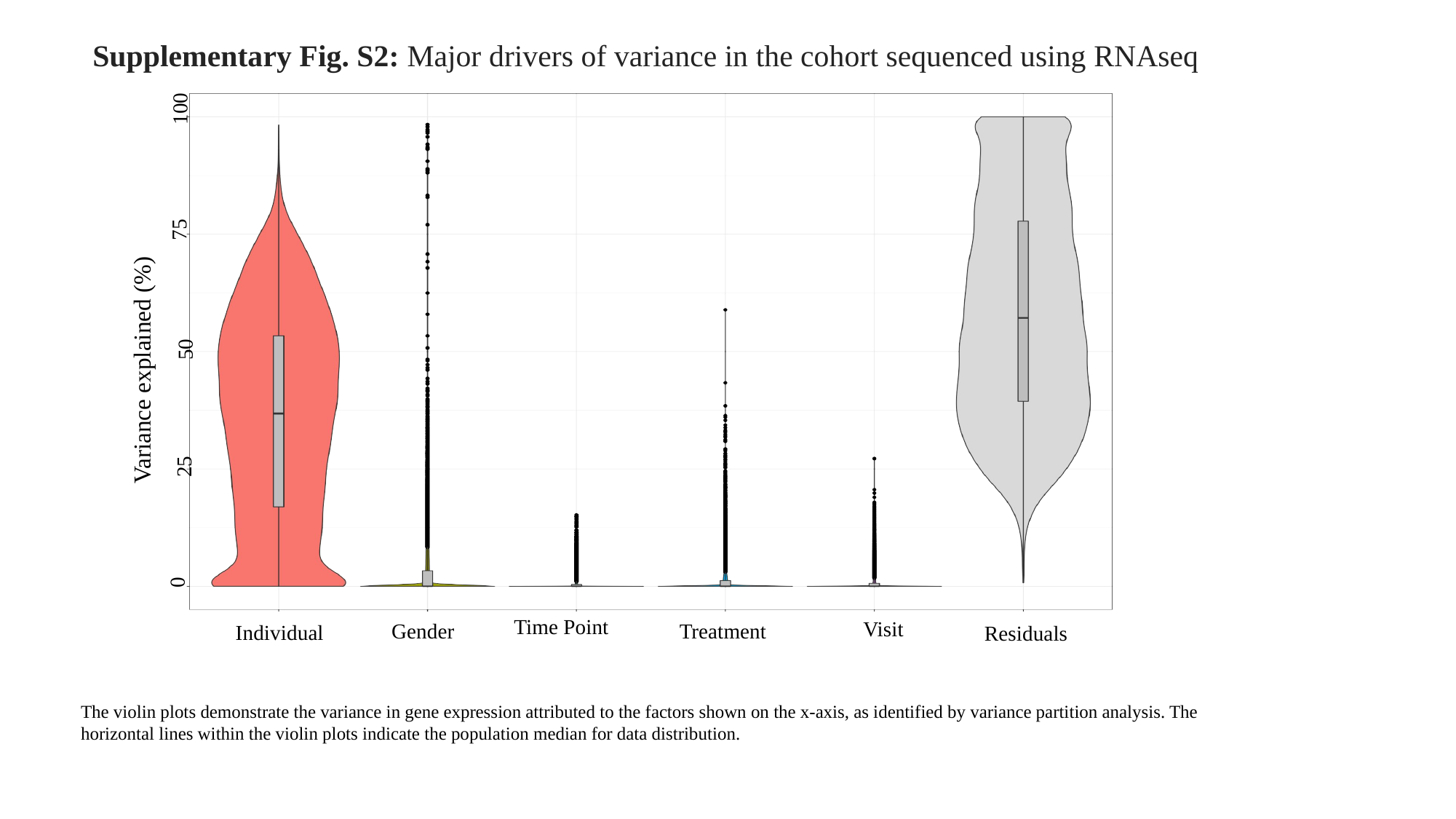

Variance explained (%)
0
75
100
25
50
Individual
Gender
Time Point
Treatment
Visit
Residuals
Supplementary Fig. S2: Major drivers of variance in the cohort sequenced using RNAseq
The violin plots demonstrate the variance in gene expression attributed to the factors shown on the x-axis, as identified by variance partition analysis. The horizontal lines within the violin plots indicate the population median for data distribution.
